# Comfort eating or toasting to your success? Self-gifting choices vary between good and bad days

**DOI:** 10.3389/fpsyg.2025.1685756

**Published:** 2026-01-21

**Authors:** Annelie J. Harvey, Suzanna E. Forwood

**Affiliations:** School of Psychology, Sport and Sensory Science, Anglia Ruskin University, Cambridge, United Kingdom

**Keywords:** alcohol, food, self-consolation, self-gifting, self-reward

## Abstract

Previous research suggests people are motivated to self-gift to either reward or console themselves. Little research has considered whether these motivations predict different types of food/alcohol or non-food self-gifting behaviors. In the current study, 280 participants were recruited online and randomly assigned to imagine either a good, bad or average (control) day at work. Participants then reported their likelihood/probability of engaging in different self-gifting behaviors (an alcoholic drink, a takeaway, a chocolate bar, an online shopping spree and a bubble bath). Relevant predictor variables (deservingness, self-esteem and the three-factor eating questionnaire) and demographic variables (age and gender) were also controlled for in the analysis. Results revealed that participants opted to self-gift food/alcohol and non-food, over and above other predictor variables, depending on the type of day they imagined. After imagining a bad (versus an average) day, participants were more likely to want to indulge in an alcoholic drink, a takeaway, a chocolate bar and a bubble bath, suggesting these items are primarily motivated by a desire for self- console. Indulging in a takeaway was the only self-gifting item that was motivated by both a desire to self-reward and self-console. The implications of these findings are discussed.

## Introduction

Self-gifting was first conceptualized by Mick and DeMoss (1990b) as “personally symbolic self-communication through special indulgences that tend to be premeditated and highly context bound” (p. 328). A self-gift can be thought of as an intentional and deliberate pleasure one allows oneself. Early research on self-gifting identified two main dimensions of the behavior: people are motivated to self-gift to reward themselves for their successes or because self-gifting acts as a type of therapy to console themselves following failure (e.g., Mick and DeMoss, 1990a, 1990b, 1992; Mick and Faure, 1998). Mick and DeMoss (1990b) described self-reward motivation as an individual perceiving themselves as deserving a treat following their achievements, whereas self-consolation is the motivation to use self-gifting to seek therapy, relief or escape from their negative situation.

Although other motivations for self-gifting exist, such as self-gifting for celebratory reasons (e.g., birthdays and holidays) and due to having surplus funds (Mick and Demoss, 1990b), the literature has predominantly considered the main motivations for self-gifting as reward and consolation. Some research suggests self-gifting is more prominent following successes than failures (e.g., Mick and Faure, 1998), whereas other research has found people are more likely to self-gift due to mood-repairing reasons (i.e., self-consolation rather than celebrating oneself) (e.g., Atalay and Meloy, 2006, 2011).

From a public health perspective, understanding self-gifting is valuable. The options for self-gift are diverse, ranging from consuming things (food, drink), sensing things (bathing, reading, music) and doing things (walking, shopping). Whilst all these options may satisfy the motivation for self-gifting, some of them may also affect the individual in other ways. For example, consuming food (e.g., takeaway, chocolate bar, alcoholic drink) may lead to intake of calories, alcohol, salt or sugar, which impact the health status of the individual and are leading causes of morbidity and mortality (Murray et al., 2013). By contrast, other self-gifting options do not have the same associated health risks and instead offer the benefit of self-care that promotes health and wellbeing (Narasimhan et al., 2019).

### Different self-gifts for different motivations

Hur and Choo (2016) specifically considered self-gifting within the attractiveness domain with female participants in their 20s and 30s. The researchers asked participants to read a scenario describing either success or failure within a job seeking context, before participants stated their preference for appearance enhancing products (e.g., lipstick) and experiences (e.g., makeup services) and problem-solving products (e.g., body shaping undergarments) and experiences (e.g., spa services). The researchers found that women showed a preference for enhancing products in the success condition and problem-solving experiences in the failure condition. Hur and Choo (2016) reason that a material product is preferred in a reward condition to enhance and serve as a reminder of the success, whereas a product would serve as an unwanted reminder for those in the failure, hence experiential options are more desirable when one is motivated to console oneself through self-gifting.

In support, David Clarke and Mortimer (2013) found an association between post-purchase regret and self-gifting when participants were self-gifting due to consolation motivations, but not when the motivation was for rewarding oneself. That is, actual products appear to be less favorable when one is seeking self-consolation compared to self-reward. When people *do* self-gift products to console themselves and repair their mood, these gifts tend to be less functional and durable (Atalay and Meloy, 2006). Therefore, it would be expected that individuals who are self-gifting in an attempt to console themselves will show a preference for either experiences or short-lived products (e.g., taking a bubble bath or food and alcohol) over long-lasting products (e.g., shopping spree).

### Self-gifting food and alcohol

When thinking of celebrating one’s success, it is not uncommon to think of “raising a glass” of champagne to “give a toast.” Alternatively, alcohol can also be described as being used to “drown your sorrows.” Similarly, one can think of delicacies to indulge in as a celebratory “treat” (e.g., steak or oysters), but also certain dishes are sometimes referred to as “comfort food.”

Research on eating and food choice gives credence to such adages. For example, stress is known to cause overeating, particularly the overeating of unhealthy foods (Hill et al., 2022). Individual differences are seen in this behavior, with more restrained individuals (e.g., chronic dieters) being more susceptible to stress-induced “comfort” eating (Greeno and Wing, 1994). Research into the link between positive mood and eating is less well established, but more recent lab studies have shown that induced positive mood can also lead to eating (Evers et al., 2013). Both findings are sufficiently robust to be seen in meta-analyses (Evers et al., 2018; Hill et al., 2022). Therefore, eating in response to stress could be considered a form of self-consolation, and eating in response to positive mood as a form of self-reward. As such, the present study examines how food and alcohol are used for self-gifting when one is seeking self-reward and/or self-consolation.

In support, previous research has suggested that people use food and beverages as a form of self-gifting. When asked to report their self-gifting purchases, a notable proportion of undergraduate student participants (20%) reported a food or beverage purchase (Atalay and Meloy, 2011). Despite this finding, however, relatively little research has considered what types of food and beverages people use for self-gifting and in response to which motivation (i.e., self-reward or self-consolation).

One exception is a study by Vassilikopoulou (2023), who conducted in-depth interviews to examine themes in the purchase of wine as a self-gift. Vassilikopoulou (2023) found evidence for consumers self-gifting wine in response to negative emotional or stressful conditions (i.e., self-consolation), as well as to reward themselves and celebrate. As such, wine, and therefore perhaps alcohol more generally, could be an appropriate self-gifting purchase that satisfies both the self-reward and self-consolation motivations.

In terms of food, Tezer and Sobol (2021) found participants will overstate their life problems to justify choosing an indulgent chocolate brownie over a healthier option (versus a condition where participants did not have a healthier option available). Essentially, participants who opted for chocolate indulgence reported more severe life problems, creating a stronger feeling of deserving the indulgent treat. In other words, individuals reasoned that the self-gift of a chocolate bar was due to consolation reasons after the fact, suggesting chocolate, at least, may be a self-gifting purchase primarily reserved for self-consolation.

### Role of demographics and other predictors

Research has already considered several different variables that predict an individual’s propensity to self-gift; primarily, age, gender, deservingness and self-esteem. Regarding age and gender, research suggests those who are younger (versus older) and female (versus male) are more likely to self-gift. Mick and DeMoss (1992) found that older people were less likely to self-gift than younger individuals and reasoned that as individuals grow older, they are satisfied with their accrued memory-laden possessions, so lack the motivation to purchase further products. Women are also reported to self-gift more than men (Ward and Tran, 2008), possibly as a function of being more active in shopping for gifts in general compared to men (Yin, 2003).

Regarding psychological variables, Malik and Vo (2024) found people were more likely to engage in self-gifting behavior if they had experienced a tumultuous rather than a harmonious breakup of a romantic relationship, and such an effect was mediated by perceived deservingness. That is, one is more likely to self-gift if they perceive themselves as deserving of consolation, ultimately justified by a motivation for self-consolation (also see Tezer and Sobol, 2021). In addition, Mick and DeMoss (1990b) emphasized the role of self-esteem in self-gifting, finding its presence as a theme in 25% of their qualitative reports. Mick and DeMoss (1990b) state that “…as messages from and to oneself, self-gifts can be elevating, protective, or medicative to self-esteem” (p. 325). Further, Bozacı (2021) reasoned that self-gifting is an individualistic behavior with a heavy self-focus and therefore found self-esteem to be a significant predictor of all forms of self-gifting.

Further psychological variables specifically predict individual differences in eating behaviors. Dietary restraint is self-control in the context of eating and is typically associated with dieting for weight loss (Karlsson et al., 2000). As previously mentioned, eating in response to stress is more prevalent in restrained individuals (Greeno and Wing, 1994; Hill et al., 2022), and therefore self-gifting food, particularly unhealthy food such as chocolate or takeaways, following a negative experience would be expected in those high in restraint. Cognitive Restraint is a subscale of the Revised 18-item Three-Factor Eating Questionnaire (Karlsson et al., 2000), the other two being Emotional Eating and Uncontrolled Eating, both of which are also plausible predictors of self-gifting food. For example, Van Strien et al. (2019) showed that palatable foods provide comfort specifically for high emotional eaters.

### Current study

Despite there being evidence of food and beverage purchases reported as self-gifts (Atalay and Meloy, 2011), research is limited in understanding the motivations people use when self-gifting food and alcohol. Typically, studies do not compare items, because only a single food or beverage is included, (i.e., wine; Vassilikopoulou, 2023), and they do not compare motivations, because only a single motivation is used (i.e., self-consolation; Atalay and Meloy, 2011; Tezer and Sobol, 2021). Studies also do not control for plausible demographic or psychological predictors of self-gifting behavior. The current study therefore sought to investigate whether different self-gifts (both food and non-food) are more likely to be used as self-gifts following self-reward or self-consolation motivations, versus a control condition, after controlling for individual psychological (self-esteem, deservingness, eating behavior for food related behaviors only) and demographic (age and gender) predictors of self-gifting behavior.

The current study manipulated self-reward, self-consolation and control motivation to self-gift by asking participants to imagine a good, bad or average day at work, following the example of Hur and Choo (2016). In contrast to previous research, this is the first study, to the authors’ knowledge, to include a range of both food and non-food self-gifts, and the options included are therefore sufficient to make an initial comparison. In line with previous research, the current study included two options for non-food self-gifting behaviors: a generic product-based option (i.e., a shopping spree) and an experiential option (i.e., a bubble bath). Previous research suggests experiential self-gifts are preferred for self-consolation and products for self-reward (e.g., Hur and Choo, 2016). In line with a wide range of research on diet (Evers et al., 2018; Hill et al., 2022), the current study included food self-gifting behaviors: both a sweet and a savory less healthy food item (i.e., a takeaway, a chocolate bar) and an alcoholic drink (i.e., an alcoholic drink). The current study has opted not to categorize these further based on their attributes – such as healthy vs. unhealthy, short vs. long lived, sustainable vs. wasteful – for two reasons. Firstly, the study does not ask participants for their preferred version of the self-gifting item, and this may alter category membership (e.g., take-away choices vary in healthiness, bubble bath choices vary in wastefulness). Secondly, more research is needed to explore which attributes, above others, are most pertinent to self-gifting behavior.

We predicted that participants would be more likely to indicate they would indulge in an alcoholic drink following both an imagined bad and good day compared to the control, in line with research on self-gifting wine both due to self-reward and self-consolation motivations (Vassilikopoulou, 2023). We predicted that participants would be more likely to indicate they would indulge in a chocolate bar in the imagined bad day condition compared to the control, in line with research from Tezer and Sobol (2021). Finally, we predicted that participants would be more likely to indicate they would indulge in a takeaway and a bubble bath following an imagined bad day compared to the control, whereas would be more likely to indicate they would indulge in a shopping spree following an imagined good day versus control, in line with research suggesting experiential self-gifts are preferred for self-consolation and products are favored for self-reward reasons (e.g., Hur and Choo, 2016).

## Methods

### Participants

A total of 311 participants from the UK were recruited online via Prolific, an online participant recruitment system, in return for payment of £2. Eight participants were excluded from further data analysis due to not completing the study. A further 22 participants were excluded from the data analysis because they failed the attention check item, which asked them to select a response that best described the day they had been previously asked to imagine (see results section for more information). Thus, the final sample included for analysis consisted of 280 participants (51% female, 49% male, 1% non-binary/third gender; *M*_age_ = 47.29 years; *SD*_age_ = 15.94). This study received ethical approval from School of Psychology, Sports and Sensory Sciences Research Committee at Anglia Ruskin University.

### Materials

#### Imagined day scenario

Three short scenarios were generated, which depicted either a good, bad or an average (control) day at work. Participants were randomly assigned to one of the conditions and told to imagine themselves being in the situation when reading the scenario. The wording for the manipulation can be found in Appendix 1.

#### Perceived deservingness

Four items were used to measure perceived deservingness of self-gifting from Cavanaugh (2014; e.g., “I feel deserving of treating myself with nice things”). Participants rated their perceived deservingness of these items on a scale from 1 = “not at all deserving” to 7 = “extremely deserving.” These items demonstrated good internal reliability (*α* = 0.95), so were combined and averaged to create an overall score of Perceived Deservingness.

#### Likelihood of self-gifting behavior

Two items were used to measure participants’ likelihood to self-gift, adapted from Malik and Vo (2024): “Following your imagined day at work, how likely/probable is it that you would indulge in any of the below for yourself?,” 1 = “very unlikely/improbable” to 7 = “every likely/probable.” Participants responded to these questions for 5 different behaviors: an alcoholic drink, a takeaway, a chocolate bar, an online shopping spree, and a bubble bath. The two likelihood/probability items demonstrated good internal reliability for each of the five behaviors (*α*’s > 0.93), so were combined and averaged to create an overall score of likelihood of self-gifting for each of the five self-gifting behaviors.

#### Self-esteem

Participants’ self-esteem was assessed via Rosenberg (1965) 10 item self-esteem scale (1 = “strongly agree” to 4 = “strongly disagree”). After reverse scoring the necessary items, this measure demonstrated good internal reliability (*α* = 0.92), so the 10 items were summed to create an overall self-esteem score, where higher values are indicative of greater self-esteem.

#### Three factor eating questionnaire (TFEQ-18)

The 18- item Three-Factor Eating Questionnaire from Karlsson et al. (2000) was included. The three distinct factors from the questionnaire each showed acceptable internal consistency, so were summed to create an overall measure of Cognitive Restraint (*α* = 0.75), Uncontrolled Eating (*α* = 0.91) and Emotional Eating (*α* = 0.88).

#### Manipulation check

To assess whether the imagined day manipulation truly prompted participants to see their hypothetical day as good/bad/average, participants were asked to “Think back to the day you imagined earlier. How would you describe the day?.” Participants responded on a continuous sliding visual analogue scale from −50 (bad) to +50 (good).

#### Attention check

To check whether participants had correctly read the previously presented imagined day scenario, participants were asked “How was the day described in the scenario you read?.” Participants chose one of four options: “Terrible,” “Fantastic,” “Average” or “Cannot remember.”

#### Exclusion item

There could be many reasons why participants would not engage with certain self-gift behaviors due to religious, financial, practical, accessibility reasons etc. For example, one may not have a bath in their home, have an aversion to making purchases online, be teetotal or live remotely with no takeaway availability. Therefore, participants were asked “In your day-to-day life, which of the following would you simply not do (for whatever reason)?.” Participants could select all of the following options that applied: “have an alcoholic drink,” “have a takeaway,” “have a chocolate bar,” “have an online shopping spree,” “have a bubble bath.”

#### Demographic questions

Participants were asked to state their age (in years) and gender for demographic reasons (male/female/non-binary/prefer not to say/prefer to describe in another way [open ended]).

### Procedure

Participants were recruited to participate in an online survey titled “Investigating Self-gifting Behavior” via Prolific. The online survey was conducted using Qualtrics. Participants were presented with a participant information sheet and provided informed consent before participating in the study.

Participants were randomly assigned to read and imagine either a good, bad or an average (control) day at work (see Appendix 1). Participants then answered questions on their perceived deservingness of rewarding themselves, followed by questions asking their likelihood/probability of engaging in the following self-gifting behaviors after their imagined day at work: an alcoholic drink, a takeaway, a chocolate bar, online shopping spree and a bubble bath. Next, participants answered the manipulation check and attention items, before answering the self-esteem scale and the three-factor eating questionnaire. Finally, participants answered demographic questions of age and gender and the exclusion item question, before they were debriefed and paid £2 via Prolific for their participation.

## Results

### Attention check

A total of 22 participants were excluded from further analysis for failing to select the correct descriptor corresponding to their imagined day allocated condition. In the good day condition, five participants incorrectly listed their day as “Average” and one participant recognized their day as “Terrible.” In the bad day condition, one participant incorrectly listed their day as “Fantastic,” two as “Average” and one said they “Couldn’t remember.” In the control condition, 10 participants listed their day as “Fantastic,” one as “Terrible” and one said they “Couldn’t remember.” After excluding these 22 participants, a total of 281 participants remained, with 94 participants in the good day condition, 89 in the control condition and 97 in the bad day condition, see Table 1.

**Table 1:** Participant characteristics by randomized group.

Characteristic	Imagined Day		
	Good Day	Control	Bad Day
*N*	94	89	97
Gender			
Male	43%	58%	43%
Female	56%	42%	54%
Non-binary or other	1%	0%	1%
Age	47.73 (16.47)	47.93 (15.33)	46.25 (16.09)
Cognitive restraint	15.12 (3.94)	15.02 (3.82)	14.74 (4.36)
Uncontrolled eating	19.53 (6.71)	18.68 (6.34)	19.75 (5.36)
Emotional eating	6.62 (2.91)	6.47 (2.69)	7.08 (2.54)
Self-esteem	30.06 (5.99)	29.64 (6.66)	28.48 (5.68)

### Manipulation check

To assess the success of our manipulation in encouraging participants to imagine a good, bad or average day, participants were asked to rate their day from bad (−50) to good (+50) on a sliding scale. Since a large sample size was used (>30 per group) and the central limit theorem applies here, a parametric one-way ANOVA was conducted to investigate if there were significant differences between the imagined group condition on responses to this manipulation check item. The ANOVA revealed a significant difference in how participants described their day according to their imagined day condition, *F* (2,278) = 1550.79, *p* < 0.001, η^2^ = 0.92. Post-hoc Tukey tests showed each comparison to be significant. Participants in the bad day condition (*M* = −41.82, *SD* = 8.69) reported their imagined day as significantly worse than participants in the good day (*M* = 46.85, *SD* = 5.80) and control conditions (*M* = 20.99, *SD* = 16.98). Participants in the good day condition rated their imagined day as significantly better than participants in the control condition (all *p* < 0.001).

### Exclusion item

Participants were excluded from the main analyses on self-gifting behaviors if the self-gifting behavior was something a participant would simply never engage with. See Table 2 to determine how many people said they would simply not engage in each self-gifting behavior, and therefore how many participants remained for analyses for each self-gifting behavior.

**Table 2:** *N* who would not engage in each self-gifting behavior and N for each self-gifting behavior analysis.

Participants	Alcoholic drink	Takeaway	Chocolate bar	Online shopping spree	Bubble bath
*N* participants who would not engage with Self-gifting behavior	109	81	25	133	143
*N* remaining participants	172	200	256	148	138

### Imagined day X self-gifting behaviors

**Figure 1:**
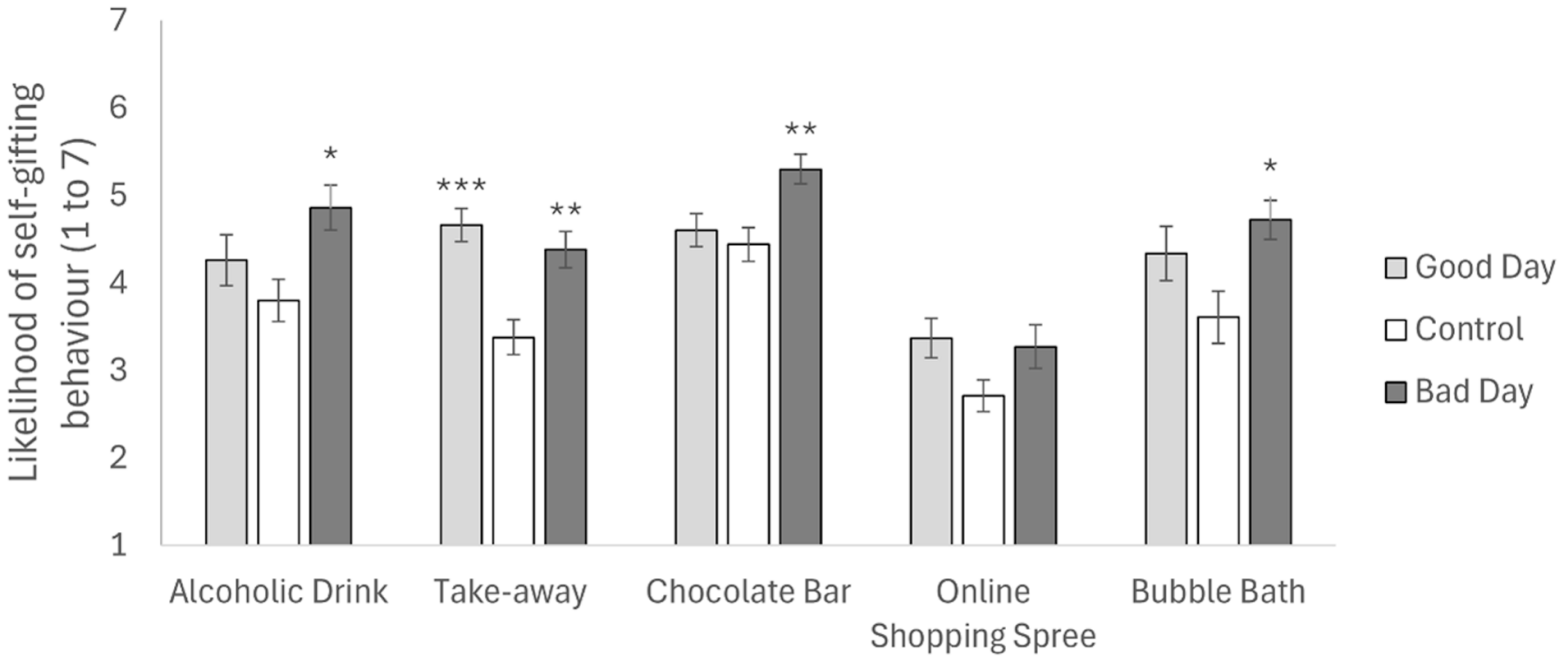
Mean reported likelihood of self-gifting behaviors by imagined day. No data is included from participants who reported they would simply never engage in the behavior, so participants vary by self-gifting behavior. Error bars indicated SEM. Stars indicate post-hoc comparisons with the control group: *p < 0.05, **p < 0.01, ***p < 0.001.

To assess the likelihood of the different self-gifting behaviors occurring in response to imagining a good, bad or average day, five one-way ANOVAs were conducted. Since a large sample size was used (>30 per group) and the central limit theorem applies here, parametric one-way ANOVA was utilized. See Figure 1: Mean reported Likelihood of self-gifting behaviors by imagined day. No data is included from participants who reported they would simply never engage in the behavior, so participants vary by self-gifting behavior. Error bars indicated SEM. Stars indicate post-hoc comparisons with the control group: * *p* < 0.05, ** *p* < 0.01, ***p < 0.001.

Table 3 and Figure 1 for the likelihood of each self-gifting behavior by imagined day.

**Table 3:** Descriptive statistics for perceived deservingness, likelihood of five self-gifting behaviors, self-esteem by imagined day condition.

Group	Perceived deservingness	Self-gifting alcohol	Self-gifting takeaway	Self-gifting chocolate bar	Self-gifting online shopping spree	Self-gifting bubble bath
Good day						
*N*	95	54	64	84	52	46
*Mean*	5.36	4.26	4.66	4.60	3.37	4.34
*SD*	1.03	2.14	1.54	1.73	1.64	2.11
Control						
*N*	89	64	68	80	48	38
*Mean*	4.01	3.80	3.38	4.44	2.71	3.61
*SD*	1.16	1.92	1.63	1.73	1.28	1.83
Bad day						
*N*	97	54	68	92	48	54
*Mean*	4.05	4.86	4.38	5.30	3.27	4.72
*SD*	1.83	1.95	1.74	1.62	1.74	1.64
Total						
*N*	281	172	200	256	148	138
*Mean*	4.48	4.28	4.13	4.80	3.12	4.29
*SD*	1.52	2.04	1.72	1.73	1.58	1.90

Imagined day had a significant impact on likelihood of self-gifting alcohol, *F* (2,169) = 4.14, *p* = 0.018, η^2^ = 0.05. Post-hoc Tukey tests showed that when compared with participants in the control condition (*M* = 3.80, *SD* = 1.92), participants in the bad day condition were significantly more likely to self-gift alcohol (*M* = 4.86, *SD* = 1.95, *p* = 0.013, Cohen’s *d* = 0.53), whereas participants in the good day condition were not significantly more likely to self-gift alcohol (*M* = 4.26, *SD* = 2.14, *p* = 0.425, Cohen’s *d* = 0.23).

Imagined day had a significant impact on likelihood of self-gifting a takeaway meal, *F*(2,197) = 11.09, *p* < 0.001, η^2^ = 0.10. *Post-hoc* Tukey tests showed, when compared with participants in the control condition (*M* = 3.38, *SD* = 1.63), both participants in the bad day condition (*M* = 4.38, *SD* = 1.74, *p* = 0.002, Cohen’s *d* = 0.60) and participants in the good day condition (*M* = 4.66, *SD* = 1.54, *p* < 0.001, Cohen’s *d* = 0.78) were significantly more likely to self-gift a takeaway meal.

Imagined day had a significant impact on the likelihood of self-gifting chocolate, *F*(2,253) = 6.54, *p* = 0.002, η^2^ = 0.05. *Post-hoc* Tukey tests showed, when compared with participants in the control condition (*M* = 4.44, *SD* = 1.73), participants in the bad day condition were significantly more likely to self-gift chocolate (*M* = 5.30, *SD* = 1.62, *p* = 0.003, Cohen’s *d* = 0.51), whereas participants in the good day condition were not significantly more likely to self-gift chocolate (*M* = 4.60, *SD* = 1.73, *p* = 0.822, Cohen’s *d* = 0.09).

Imagined day had no significant impact on likelihood of self-gifting an online shopping spree, *F* (2,145) = 2.52, *p* = 0.018, η^2^ = 0.03. *Post-hoc* Tukey tests showed, when compared with participants in the control condition (*M* = 2.71, *SD* = 1.28), neither participants in the bad day (*M* = 3.27, *SD* = 1.74, *p* = 0.187, Cohen’s *d* = 0.36), nor participants in the good day condition (*M* = 3.37, *SD* = 1.64, *p* = 0.094, Cohen’s *d* = 0.42) were more likely to self-gift an online shopping spree.

Imagined day had a significant impact on likelihood of self-gifting a bubble bath, *F*(2,135) = 4.04, *p* = 0.020, η^2^ = 0.06. *Post-hoc* Tukey tests showed, when compared with participants in the control condition (*M* = 3.61, *SD* = 1.83), participants in the bad day condition were significantly more likely to self-gift a bubble bath (*M* = 4.72, *SD* = 1.64, *p* = 0.015, Cohen’s *d* = 0.60), whereas participants in the good day condition were not significantly more likely to self-gift a bubble bath (*M* = 4.34, *SD* = 2.11, *p* = 0.176, Cohen’s *d* = 0.39).

### Predictors of self-gifting

Hierarchical regression models were conducted to address the question of whether demographics, deservingness, self-esteem and dietary behaviors predict self-gifting behaviors, and whether imagined day predicts self-gifting above and beyond these demographics or trait measures. For each of the self-gifting behaviors, a two-stage hierarchical regression (enter method) was carried out. In each analysis, age and gender (dummy coded, reference male), deservingness and self-esteem were entered in step one, with the eating behaviors also included where self-gifting was a food item, and imagined day (dummy coded, reference control) entered at step two. These regression results are presented in Table 4.

**Table 4:** Results of two-step hierarchical multiple regressions predicting self-gifting behaviors with regression coefficients (B) specified for all predictor variables at each step of the regression.

Predictor variables	Self-gifting alcohol	Self-gifting takeaway	Self-gifting chocolate bar	Self-gifting shopping spree	Self-gifting bubble bath
Step 1					
Intercept	3.16***	1.53	3.37***	1.40	3.06**
Age	0.01	−0.01	0.00	−0.01	0.01
Gender	−0.31	0.01	−0.09	0.28	0.93**
Deservingness	0.13	0.42***	0.19**	0.51***	0.17
Self-esteem	0.01	0.03	0.01	0.49	−0.02
Cog restraint	–	−0.05	−0.05	–	–
Uncontrolled E	–	0.05	0.01	–	–
Emotional E	–	0.02	0.15**	–	–
*R* ^2^	0.02	0.20	0.12	0.23	0.09
Step 2					
Intercept	2.54***	1.09	2.69***	1.06	2.15
Age	0.01	−0.01	0.00	−0.01	0.01
Gender	−0.37	−0.07	−0.12	0.24	0.89**
Deservingness	0.13	0.38***	0.26***	0.52***	0.19
Self-esteem	0.02	0.03	0.01	−0.01	−0.01
Cog restraint	–	−0.05	−0.04	–	–
Uncontrolled E	–	0.05	0.02	–	–
Emotional E	–	0.02	0.14*	–	–
Imagined day					
Good (ref C)	0.45	0.89**	−0.21	0.11	0.51
Bad (ref C)	1.18 **	1.00***	0.80**	0.46	1.00*
*R* ^2^	0.08	0.27	0.18	0.25	0.14
Δ*R*^2^	0.06	0.07	0.06	0.01	0.05
*F*	4.97***	8.37***	8.54***	1.32	3.37*

*p < 0.05; **p < 0.01, ***p < 0.001.

This analysis revealed two findings. Firstly, relatively few predictors of self-gifting behavior emerge from Step 1. Deservingness predicts self-gifting behaviors of indulging with a takeaway, a chocolate bar and a shopping spree only. Gender predicts only the self-gift of taking a bubble bath, with females more likely than males to do this. Emotional eating predicts only the self-gifting behavior of eating a chocolate bar. Self-esteem, cognitive restraint and uncontrolled eating do not predict any self-gifting behaviors. This pattern remains in Step 2, when imagined day is included in the analysis. This observation shows that these factors are associated with self-gifting behavior independently of the imagined day manipulation.

Secondly, the impact of imagined day on self-gifting behavior mirrors that observed in the ANOVA analyses already reported: with imagined good days increasing the likelihood of self-gifting a takeaway and imagined bad days increasing the likelihood of self-gifting alcohol, takeaway, chocolate and a bath. As this pattern remains significant even with covariates, it can be concluded that imagined day impacts self-gifting behaviors independently of deservingness, gender or emotional eating.

## Discussion

The current study is novel in its attempts to consider whether a motivation for self-reward or self-consolation promotes self-gifting of a variety of food and non-food items, over and above psychological and demographic variables associated with self-gifting. Results revealed that when participants imagined a bad day at work, and therefore were motivated to console themselves, they were more likely to indulge in a bubble bath, a chocolate bar and an alcoholic drink compared to participants who had imagined an average day at work. Indulging in a takeaway was the only self-gifting option that served to satisfy both a motivation to self-reward (following an imagined good day at work) and self-console (following an imagined bad day at work). There was no difference between conditions in the likelihood of engaging in an online shopping spree. Interestingly, these effects remained, even when controlling for variables that previous research suggests are associated with self-gifting behavior (i.e., age, gender, self-esteem and deservingness) or eating behavior (i.e., measures of cognitive restraint, uncontrolled eating and emotional eating).

The findings largely support initial predictions and previous research. Previous research suggested experiences or short-lived products are preferrable for self-gifting when one is in search of self-consolation, compared to more durable products (e.g., Atalay and Meloy, 2006; Hur and Choo, 2016). In support, the present results show the likelihood of indulging with a takeaway, a chocolate bar, an alcoholic drink and a bubble bath (all experiential or short lived products) is higher for participants who had imagined a bad day compared to those who had imagined an average day at work. Specifically, regarding the self-gift of a chocolate bar following an imagined bad day at work, the findings are also supportive of those of Tezer and Sobol (2021), who found participants retrospectively justified indulging in a chocolate bar by overstating their previous hardships.

However, some of the findings differ slightly from initial predictions and previous research. Firstly, previous qualitative research on the self-gifting of wine suggested such a drink is self-gifted both for reasons of self-reward and self-consolation (Vassilikopoulou, 2023). However, the current study only found support for the latter motivation when self-gifting alcohol. The difference in findings between the current study and that of Vassilikopoulou (2023) are notably that the present study utilized an experimental quantitative design considering a generic “alcoholic drink,” whereas Vassilikopoulou (2023) focused specifically on wine within qualitative interviews. It may be that “wine” prompts people to think of celebrations (Dunn, 2016), whereas the generic wording of “alcoholic drink” in the current study does not have the same connotations. Alternatively, it could be that alcoholic drinks for self-reward are reserved for other contexts beyond simply a good day at work. Future research could consider different variations of alcoholic drinks (e.g., wine, champagne, beer) and following which types of rewarding contexts they are sought after (e.g., weddings, parties, holidays).

Secondly, no differences were found between conditions for participants engaging in an online shopping spree. That is, participants who had imagined an average day at work were just as likely as those who had imagined a good or bad day at work to engage in an online shopping spree. An online shopping spree was included as a generic option for any durable product purchase as a form of self-gifting, but this option may have been too generic. Online shopping is a commonplace occurrence in modern society, which accelerated during the COVID-19 pandemic (Young et al., 2022). The pandemic led to a rise of online shopping, even for the mundane, everyday purchase of groceries, because people valued the perceived usefulness and ease-of-use of online shopping, among other reasons (Tyrväinen and Karjaluoto, 2022). As such, an online shopping spree could have been perceived as an everyday occurrence that people would engage in after any type of day at work, and therefore not necessarily meeting the definition of a self-gift as a “special indulgence” (Mick and Demoss, 1990b, p. 328). In support, we specifically asked participants “…how likely/probable is it that you would indulge in any of the below…” and the means for participants in each experimental condition self-gifting an online shopping spree in our study were all below the midpoint of 4 (see Table 3), suggesting a low uptake in indulging in an online shopping spree generally.

Thirdly and finally, indulging in a takeaway was the only self-gifting option in our study that was likely for *both* self-reward (i.e., imagined good day) and self-consolation (i.e., imagined bad day) reasons. This non-durable product was expected for participants in the imagined bad day condition, based on previous research (e.g., Atalay and Meloy, 2006), but interestingly was also deemed a suitable self-gift for participants to reward themselves. Reflecting on this finding, the takeaway option was an indulgence which also had the addition of removing a daily chore of preparing and cooking one’s dinner, whereas all other food and drink self-gifting options could be deemed as a simple addition to one’s diet. Perhaps the double benefit of a desired meal, whilst removing the chore of preparing it, is particularly desirable after one has exerted themselves after a successful or terrible day at work. Future research could consider if there is any credence in such speculation and determine if a takeaway is a desired option following other manipulations of success and failure that are perhaps not associated with one’s personal effort (e.g., winning a raffle).

### Predictors and demographics

The role of predictors and demographics, identified as relevant from previous research, provided limited predictive contributions to the self-gifting measures. Firstly, despite early research suggesting that self-gifting declines with age (Mick and DeMoss, 1992), there was no evidence for age significantly predicting any of the self-gifting behaviors. Mick and DeMoss (1992) suggested that older individuals do not have the motivation to self-gift products due to already amassing many products in their lifespan. However, this lack of motivation to purchase products is unlikely to apply to self-gifting food/alcohol or taking a bubble bath, as such behaviors are either experiential or short-lived products. As such, it is perhaps not surprising that age is not a predictor of experiential self-gifting in the current study, but age cannot be discounted as a negative predictor when one is self-gifting more durable products.

Secondly, although some previous research emphasized the role self-esteem plays within self-gifting (Mick and Demoss, 1990b), our results supported Atalay and Meloy’s (2006) previous findings where no relationship was identified between self-esteem and self-gifting. Puzzlingly, the current study identified that perceptions of deservingness of self-gifts predicted some self-gifting behaviors (i.e., a takeaway, a chocolate bar and an online shopping spree), but not others (i.e., an alcoholic drink or a bubble bath). Previous research by Malik and Vo (2024) suggested that deservingness played a mediating role between whether individuals had experienced a tumultuous breakup of a romantic relationship (versus harmonious) and self-gifting. As such, the relationship between deservingness and self-gifting might be expected to diminish when accounting for participants’ imagined bad days in the regression model, but this was not the case. Further research could explore when and why people feel deserving of particular self-gifts, perhaps via qualitative methods to gain an in-depth perspective of people’s thought processes and justifications.

Regarding gender, we found that self-gifting food and alcohol did not differ between male and female participants. Although research suggests women purchase gifts (Yin, 2003) and self-gift more than men (Ward and Tran, 2008), perhaps food and alcohol are unique purchases that are engaged with equally across genders. In support, research suggests that although overall alcohol consumption is greater in men than women, female alcohol consumption is consistently rising (Moinuddin et al., 2016), and therefore self-gifting oneself an alcoholic drink could be appealing to both men and women equally. Gender was a significant predictor in the self-gifting behavior of taking a bubble bath however, where women were more likely to engage in this behavior than men. Such a finding is in line with research from the health literature, which finds women are more likely than men to engage in self-care activities for a healthy lifestyle (e.g., Grzywacz et al., 2012).

The current findings only partially confirm previous research on the link between cognitive restraint or emotional eating and self-gifting. While emotional eating was associated with self-gifting of a chocolate bar, aligning with research on comfort eating in this group (van Strien et al., 2019), there were no associations with cognitive restraint, even though the role of restraint in stress-induced eating is well established (Evers et al., 2018; Hill et al., 2022). One reason for this is the gap between stress induction in lab studies and the induced ‘Good day’ or ‘Bad day’ conditions utilized in the current study. While imagining a bad day at work would elicit some of the affective and cognitive aspects of stress, it is less likely to generate the physiological aspects those experiences would generate if actually experienced. Therefore, cognitive restraint may play a larger role in predicting self-gifting of food items in more impactful, stressful situations.

It is noteworthy that the relationships that were identified between the predictors and self-gifting behaviors did not change in significance when the type of day participants had previously imagined was controlled for. In other words, gender, deservingness and emotional eating are predictors of their corresponding self-gifting behaviors, regardless of whether participants are pursuing a self-reward or self-consolation motivation for self-gifting.

### Limitations

The main limitation of the current study was that the manipulation for self-reward or self-consolation motivated self-gifting and the self-gifting choices were hypothetical. As such, predicted behavior following an imagined good or bad day at work was measured, rather than participants’ actual self-gifting behavior or mood or motivation for making their choice. This raises several limitations. Firstly, it is assumed that self-gifting is motivated by self-consolation following a bad day and self-reward following a good day, and that mood varies with the imagined day. Future research would need to measure motivation and mood to confirm this. Future research also could adopt different methodologies to measure actual self-gifting behavior of food and drink, such as asking participants to keep a diary of their self-gifting experiences (see Atalay and Meloy, 2011). Alternatively, Ecological Momentary Assessment (EMA) is a tool that periodically prompts participants to report their behavior via electronic reminders to capture real time reports of people’s behavior in their natural environments (Shiffman et al., 2008). Such a tool could seek to confirm if the pattern of the hypothetical self-gifting preferences for food and alcohol identified in the current study is mirrored in actual behavior.

In addition, the self-gifting choices utilized in this study were generic in places (i.e., an alcoholic drink, an online shopping spree). A such, online shopping for products as a form of self-gifting following an imagined bad/good day at work cannot be discounted, especially as other research has found evidence for specific products being self-gifted due to specific motivations (e.g., headphones for self-consolation, Malik and Vo, 2024). Similarly, by being more specific about the types of possible self-gifts available, a different pattern of results when considering food and drink may have been observed. As mentioned previously, wine or champagne may be specifically associated with self-reward and celebration (Dunn, 2016), whereas other alcoholic drinks might not. Therefore, future research could build on the current findings further by utilizing more specific (e.g., wine, beer, champagne) rather than general categories (i.e., an alcoholic drink) to gain a more in-depth understanding of self-gifting behavior regarding food and beverages.

### Implications

The current findings can inform future interventions designed to redirect self-gifting behaviors away from unhealthy food choices (e.g., takeaway, chocolate bar, alcoholic drink) and towards healthier options (i.e., bubble bath). The current study revealed that participants who had imagined a bad day (compared to those in the control condition) were more likely to self-gift themselves a takeaway, chocolate bar, alcoholic drink and a bubble bath. Presuming one self-gift is enough to satisfy the self-consolation motivation, future research could consider how, when and why people are prompted to opt for self-care over unhealthy food and drink choices. In extremes, unhealthy eating can cause health issues and strain the national health service (Murray et al., 2013), whereas self-care is considered of great importance to general health and wellbeing (Narasimhan et al., 2019). As such, public health messages and campaigns could target those who are experiencing failure or are in need of consoling with messages prompting self-care, grooming and pampering to promote this type of healthy experiential self-gifting behavior over unhealthy food and drink choices.

## Data Availability

The datasets presented in this study can be found in online repositories. The names of the repository/repositories and accession number(s) can be found at: https://osf.io/z2xjh/.

## Appendix 1

Wording, and associated instructions, for the Imagined Day scenarios.

Please read the following scenario and imagine YOURSELF being in this situation:

**Table:**

Good Day	Control	Bad Day
You have had a fantastic day at work. Everything has gone right. You met two deadlines, had a productive meeting with your manager and completed your long “to do” list. You feel energized and content.	You have had an average day at work. Everything has gone as expected. You set two deadlines, had a routine meeting with your manager and made some progress through your “to do” list. You feel normal and average.	You have had a terrible day at work. Everything has gone wrong. You missed two deadlines, had a tense meeting with your manager and failed to complete your long “to do” list. You feel depleted and miserable.
